# Tea consumption and long-term risk of type 2 diabetes and diabetic complications: a cohort study of 0.5 million Chinese adults

**DOI:** 10.1093/ajcn/nqab006

**Published:** 2021-03-11

**Authors:** Jia Nie, Canqing Yu, Yu Guo, Pei Pei, Lu Chen, Yuanjie Pang, Huaidong Du, Ling Yang, Yiping Chen, Shichun Yan, Junshi Chen, Zhengming Chen, Jun Lv, Liming Li

**Affiliations:** Department of Epidemiology and Biostatistics, School of Public Health, Peking University, Beijing, China; Department of Epidemiology and Biostatistics, School of Public Health, Peking University, Beijing, China; Peking University Center for Public Health and Epidemic Preparedness and Response, Beijing, China; Chinese Academy of Medical Sciences, Beijing, China; Chinese Academy of Medical Sciences, Beijing, China; Department of Epidemiology and Biostatistics, School of Public Health, Peking University, Beijing, China; Department of Epidemiology and Biostatistics, School of Public Health, Peking University, Beijing, China; Peking University Center for Public Health and Epidemic Preparedness and Response, Beijing, China; Medical Research Council Population Health Research Unit, Nuffield Department of Population Health, University of Oxford, Oxford, United Kingdom; Clinical Trial Service Unit and Epidemiological Studies Unit (CTSU), Nuffield Department of Population Health, University of Oxford, Oxford, United Kingdom; Medical Research Council Population Health Research Unit, Nuffield Department of Population Health, University of Oxford, Oxford, United Kingdom; Clinical Trial Service Unit and Epidemiological Studies Unit (CTSU), Nuffield Department of Population Health, University of Oxford, Oxford, United Kingdom; Medical Research Council Population Health Research Unit, Nuffield Department of Population Health, University of Oxford, Oxford, United Kingdom; Clinical Trial Service Unit and Epidemiological Studies Unit (CTSU), Nuffield Department of Population Health, University of Oxford, Oxford, United Kingdom; NCDs Prevention and Control Department, Heilongjiang CDC, Heilongjiang, China; China National Center for Food Safety Risk Assessment, Beijing, China; Clinical Trial Service Unit and Epidemiological Studies Unit (CTSU), Nuffield Department of Population Health, University of Oxford, Oxford, United Kingdom; Department of Epidemiology and Biostatistics, School of Public Health, Peking University, Beijing, China; Peking University Center for Public Health and Epidemic Preparedness and Response, Beijing, China; Key Laboratory of Molecular Cardiovascular Sciences (Peking University), Ministry of Education, Beijing, China; Department of Epidemiology and Biostatistics, School of Public Health, Peking University, Beijing, China; Peking University Center for Public Health and Epidemic Preparedness and Response, Beijing, China

**Keywords:** tea consumption, type 2 diabetes, diabetic complications, death, prospective cohort study, China Kadoorie Biobank

## Abstract

**Background:**

Evidence from epidemiological studies remains inconsistent or limited about the associations of tea consumption with incident diabetes and risk of diabetic complications and death among patients with diabetes.

**Objectives:**

We aimed to investigate the associations of tea consumption with long-term risk of developing type 2 diabetes (T2D) and risks of diabetic complications and death among patients with diabetes.

**Methods:**

This study included 482,425 diabetes-free participants and 30,300 patients with diabetes aged 30–79 y at study enrollment from the China Kadoorie Biobank. Tea consumption information was collected at baseline by interviewer-administered questionnaires. Incidences of diabetes, diabetic complications, and death were identified by linkages to the National Health Insurance system, disease registries, and death registries. Cox proportional hazard regression models were used to estimate HRs and 95% CIs.

**Results:**

The mean ± SD age of participants free of diabetes was 51.2 ± 10.5 y and 41% were male. The mean ± SD age of patients with diabetes was 58.2 ± 9.6 y and 39% were male. Of all daily tea consumers, 85.8% preferred green tea. In the diabetes-free population, 17,434 participants developed incident T2D during 11.1 y of follow-up. Compared with participants who never consumed tea in the past year, the HR (95% CI) of T2D for daily consumers was 0.92 (0.88, 0.97). In patients with diabetes, we identified 6572 deaths, 12,677 diabetic macrovascular cases, and 2441 diabetic microvascular cases during follow-up. Compared with patients who never consumed tea in the past year, the HRs (95% CIs) of all-cause mortality and risk of microvascular complications for daily consumers were 0.90 (0.83, 0.97) and 0.88 (0.78, 1.00), respectively. Tea consumption was not associated with risk of macrovascular complications among patients with diabetes. With regard to tea consumed, the inverse associations between daily tea consumption and risks of T2D and all-cause mortality in patients with diabetes were only observed among daily green tea drinkers.

**Conclusions:**

In Chinese adults, daily green tea consumption was associated with a lower risk of incident T2D and a lower risk of all-cause mortality in patients with diabetes, but the associations for other types of tea were less clear. In addition, daily tea consumption was associated with a lower risk of diabetic microvascular complications, but not macrovascular complications.

## Introduction

Diabetes mellitus is a major public health concern that has reached an alarming level. The overall standardized prevalence of diabetes was 12.8% in Chinese adults, reported by a nationally representative cross-sectional study in 2017 (1). Impaired glucose metabolism is a typical characteristic of diabetes, affecting multiple organ systems and leading to various diabetic complications and premature death (2–4). Tea is a type of popular beverage consumed across the world (5). The bioactive substances in tea, such as catechin, tea theaflavins, and caffeine, may play a role in enhancing insulin activity, ameliorating insulin resistance, activating the insulin-signaling pathway, protecting islet B cells, scavenging free radicals, and decreasing inflammation (6–9).

So far, evidence for tea consumption and type 2 diabetes (T2D) from epidemiological studies remains inconsistent. A meta-analysis included 14 cohort studies (10), of which 10 were conducted in Western countries (11–20) and 4 in Asian countries (21–24), reported that tea consumption could be linear inversely associated with T2D risk. Studies conducted in Europe and North America showed that tea consumption was associated with a lower risk of T2D, but a dose–response relation was only observed among European cohorts. In contrast, no clear association was found among Asian cohorts. Furthermore, only a limited number of these studies investigated specific types of tea and found an inverse association for oolong and black tea with T2D but no significant association for green tea. However, a recent publication from a cohort study conducted in Shanghai, China, reported that green tea drinking was associated with an increased risk of T2D in urban adults (25). The researchers explained that the excess risk might be because of the pesticide residue in tea leaves. In addition, there is little evidence from cohort studies focusing on the association between tea consumption and risk of diabetic complications or death among patients with diabetes.

Therefore, we aimed to investigate the associations of tea consumption with long-term risk of developing T2D and risk of diabetic complications and death among patients with diabetes based on a large Chinese cohort.

## Methods

### Study population

The design of the China Kadoorie Biobank (CKB) and baseline characteristics of the participants were described previously (26, 27). Briefly, the CKB study enrolled 512,725 participants aged 30 to 79 y from 10 areas across China (5 urban areas and 5 rural areas) during 2004–2008. Trained staff conducted face-to-face interviews using an electronic questionnaire and standard physical measurements after participants signed an informed consent. The study was approved by the ethical committees of the Chinese Center for Disease Control and Prevention and the University of Oxford.

For the analysis of the associations between tea consumption and risk of incident diabetes (**Supplemental Figure 1**), we excluded participants who had diabetes (*n* = 30,300) at baseline, which was defined as *1*) self-reported diabetes diagnosed by the county-level hospital and above or *2*) measured fasting blood glucose (FBG) of ≥7 mmol/L, or *3*) postprandial blood glucose of ≥11 mmol/L. We further excluded participants with a prior diagnosis of major chronic diseases including cancer (*n* = 2577), coronary heart disease (*n* = 15,472), or stroke (*n* = 8884), and those with missing BMI values (*n* = 2) at baseline. After these exclusions, a total of 461,074 participants were included for this analysis.

For the analysis of the association between tea consumption and risk of death and diabetic microvascular complications, we only included those who had diabetes (*n* = 30,300) at baseline; for diabetic nephropathy, we further excluded those who had chronic kidney disease (*n* = 651) at baseline. For the analysis of secondary macrovascular disease, patients with diabetes who had cancer (*n* = 294), coronary heart disease (*n* = 2699), or stroke (*n* = 1537) at baseline were excluded, leaving 26,162 patients with diabetes available for this analysis.

### Assessment of tea consumption

Information on tea consumption was assessed by the following questions:

1. During the past 12 months, how often did you drink any tea (never or rarely, only occasionally, only at certain seasons, every month but less than weekly, usually at least once a week)?
  For those who answered “usually at least once a week,” we further asked,
2. During the past 12 months, on how many days did you drink tea in a typical week (1–2 d/wk, 3–5 d/wk, daily or almost every day)?
3. At about what age did you start drinking tea almost weekly?
4. Which kind of tea do you drink mostly (green/jasmine tea, oolong tea, black tea, other tea), and how many cups do you usually drink on days when you drink tea (with 300 mL as a standard cup size)?
5. How often do you change tea leaves during a day?
6. How much tea leaves do you usually add each time (with a picture showing the amount of tea leaves in grams)?
  For those who answered other than “usually at least once a week” to question 1, we then asked:
7. In the past, did you ever have a period of at least 1 year during which you usually drank tea at least once a week?

Based on these questions, we divided participants into 3 frequency groups: those who never drank tea in the past year, those who drank tea less than daily, and current daily drinkers. For daily drinkers, we further categorized participants according to the amount of tea leaves consumed (≤2.0, 2.1–4.0, and ≥4.1 g/d), duration of consumption (<10, 10–29, and ≥30 y), and types of tea consumed (green tea and others).

### Assessment of covariates

Covariate information for all participants was collected by baseline questionnaires, including sociodemographic characteristics, lifestyle behaviors, and personal and family medical history. We quantified the level of total physical activity by summing metabolic equivalent task (MET)-hours of all activities with hours spent on specific activities as weights. Dietary intake in the past year was assessed by a validated qualitative food-frequency questionnaire (28). Trained staff took physical measurements, including height, weight, waist circumference (WC), and blood pressure, according to standard measurement procedures. A random blood sample was collected (with the time since last taking foods or drinking beverages recorded) from each participant for long-term storage, and the plasma glucose concentration was measured on-site using SureStep Plus meters (Lifescan; Johnson & Johnson). Participants with on-site random plasma glucose concentration of >7.8 mmol/L were invited for a fasting plasma glucose test the following day if they did not have a prior diabetes diagnosis.

### Ascertainment of study outcomes

The outcomes of participants were ascertained through the local Disease Surveillance Points system, disease and death registries, and the National Health Insurance (HI) system since enrollment. In addition, community workers or doctors helped check the status of participants who did not join the HI plan or who were not linked to the HI system annually. Any deaths or diseases occurring among participants were coded using the 10th revision of the International Classification of Diseases (ICD-10) by trained medical staff who were blinded to baseline information.

In the analysis of tea consumption and risk of T2D, we included incident diabetes cases (ICD-10: E11 and E14) as the endpoint outcomes and excluded other diabetes cases (ICD-10: E10, E12, and E13). We assumed that the endpoint outcomes as defined were mostly T2D because participants in this cohort were aged mostly >40 y old at baseline. Therefore, the possibility of outcome misclassification was low.

All-cause and cause-specific mortality were included as study outcomes for patients with diabetes at baseline. The underlying causes of death were classified as cardiovascular diseases, T2D, and others (**Supplemental Table 1**). The outcomes of incident diabetic complications included macrovascular (ischemic heart disease, stroke, and other macrovascular diseases) and microvascular (retinopathy, nephropathy, and neuropathy) complications.

### Statistical analyses

We counted person-years at risk for each participant from baseline (2004–2008) to the date of outcome diagnosis, death, loss to follow-up, or 31 December 2017, whichever occurred first. Cox proportional hazard models were used to quantify the associations between tea consumption and outcomes of interest, estimating HRs and their 95% CIs. The models used chronological age as the underlying time scale and were stratified jointly by sex, 10 regions, and age at baseline in 5-y intervals.

For the analysis between tea consumption and risk of incident T2D, we used participants who never consumed tea in the past year as the reference group. The multivariable models were adjusted for age (years); education (no formal school, primary school, middle school, high school, college, or university or higher); family history of diabetes (yes or no); smoking (never smoker, former smokers who have quit for reasons other than illness, current smokers, or former smokers who have quit because of illness: 1–9, 10–19, 20–29, or ≥30 cigarettes or equivalent/d); alcohol intake (nonweekly drinker; former weekly drinker; weekly drinker; daily drinker: <15, 15–29, 30–59, or ≥60 g/d of pure alcohol); level of physical activity (MET-h/d); intakes of red meat, fresh vegetables, and fruits (days/week; calculated by assigning participants to the midpoint of their consumption category); BMI (kg/m^2^); WC (centimeters); and prevalent hypertension (yes or no). For the analysis between tea consumption and risk of diabetic macrovascular complications, the models were adjusted for age; education; random glucose (millimoles/liter); treatment for diabetes (taking insulin and/or oral hypoglycemic drugs, or no treatment); smoking; alcohol intake; level of physical activity; intakes of red meat, fresh vegetables, and fruits; BMI; WC; and prevalent hypertension. For microvascular complications or mortality, we further adjusted for the prevalence of cancer, stroke, and coronary heart disease (yes or no).

We also examined the associations between tea consumption and outcomes of interest according to the duration of tea consumption or the kind of tea consumed by comparing daily consumers with those who never consumed tea. Linear trend tests were only performed in daily tea consumers by assigning the median values of tea leaves consumed or duration of consumption to each category and including them as continuous variables in models. Further, we conducted subgroup analyses according to baseline characteristics (age, sex, region, education, smoking, alcohol consumption, fresh fruit consumption, physical activity, BMI, WC, prevalent hypertension, and family history of diabetes). Likelihood ratio tests were used to compare models with and without a cross-product term.

We used Stata (version 15.0; StataCorp) for statistical analyses and R statistic software (version 4.0.2; R Foundation for Statistical Computing) for plots. All *P* values were 2-sided and statistical significance was defined as *P* < 0.05.

## Results

Of 461,047 participants without diabetes, 26.4% reported daily tea consumption with a median of 4.0 (IQR: 2.0–6.0) g of tea leaves consumed per day in urban areas and 3.0 (IQR: 2.0–4.0) g/d in rural areas. With increasing drinking frequency and tea leaves consumed, participants were more likely to be male, current smokers, and alcohol drinkers, and their WC was slightly larger (**Table 1**). Of all daily consumers, 85.8% preferred green tea. The baseline characteristics of diabetic participants categorized by tea drinking frequency were similar to participants without diabetes (**Supplemental Table 2**). In addition, daily tea consumers had a higher random glucose concentration than other participants.

**TABLE 1:** Baseline characteristics of participants without diabetes according to tea consumption^1^

	Never in the past year			Daily consumption		
		Less than daily	Daily (all)	≤2.0 g/d	2.1–4.0 g/d	≥4.1 g/d
No. of participants (%)	160,213 ± 34.8	179,282 ± 38.9	121,552 ± 26.4	46,688 ± 10.1	43,268 ± 9.4	31,596 ± 6.9
Female, %	77.4	58.4	35.6	45.5	36.0	20.3
Age, y	52.4 ± 12.0	49.6 ± 8.5	52.1 ± 10.5	52.5 ± 10.8	52.2 ± 10.4	51.3 ± 10.7
Urban, %	41.9	44.6	39.3	34.1	37.4	49.6
Middle school or higher, %	43.9	51.4	53.2	52.4	53.9	53.5
Current smoker,^2^ %						
Male	56.6	64.1	77.3	73.4	76.4	81.9
Female	2.2	2.6	4.5	4.1	4.9	6.4
Weekly or daily alcohol drinker, %						
Male	22.7	32.2	41.4	39.5	41.7	42.8
Female	1.3	2.3	5.2	4.6	5.4	7.5
Physical activity, MET-h/d	22.2 ± 13.2	22.3 ± 12.3	20.9 ± 13.6	20.9 ± 12.7	20.9 ± 12.7	20.7 ± 12.6
Regular consumption ≥4 d/wk, %						
Red meat	44.0	47.3	49.6	49.4	48.2	51.8
Fresh vegetables	98.0	98.1	98.8	98.9	98.8	98.6
Fresh fruits	23.7	28.6	32.2	32.8	31.7	31.8
Physical measurements						
BMI, kg/m^2^	23.3 ± 3.6	23.6 ± 3.4	23.8 ± 3.5	23.7 ± 3.5	23.8 ± 3.3	23.9 ± 3.4
WC, cm	79.0 ± 10.1	80.0 ± 9.3	80.4 ± 10.3	80.2 ± 9.6	80.4 ± 9.7	80.9 ± 9.5
Family history of diabetes, %	5.5	6.6	6.8	6.5	6.8	7.3
Prevalent hypertension, %	31.7	31.7	34.1	33.4	34.4	34.7
Postmenopausal women, %	49.7	48.9	48.4	48.7	48.2	48.0
Tea consumption						
Duration of consumption, y	—	—	25.3 ± 9.4	24.3 ± 9.7	25.1 ± 9.6	26.9 ± 9.8
Green tea consumers, %	—	—	85.8	85.9	85.9	85.5

1 Values are means ± SDs or percentages and were adjusted for age, sex, and region, where appropriate, using either multiple linear regression (for continuous outcomes) or logistic regression (for binary outcomes); *n* = 461,047. MET, metabolic equivalent task; WC, waist circumference.

2 Former smokers who had stopped smoking for illness were categorized as current smokers.

### Tea consumption and risk of T2D

**FIGURE 1:**
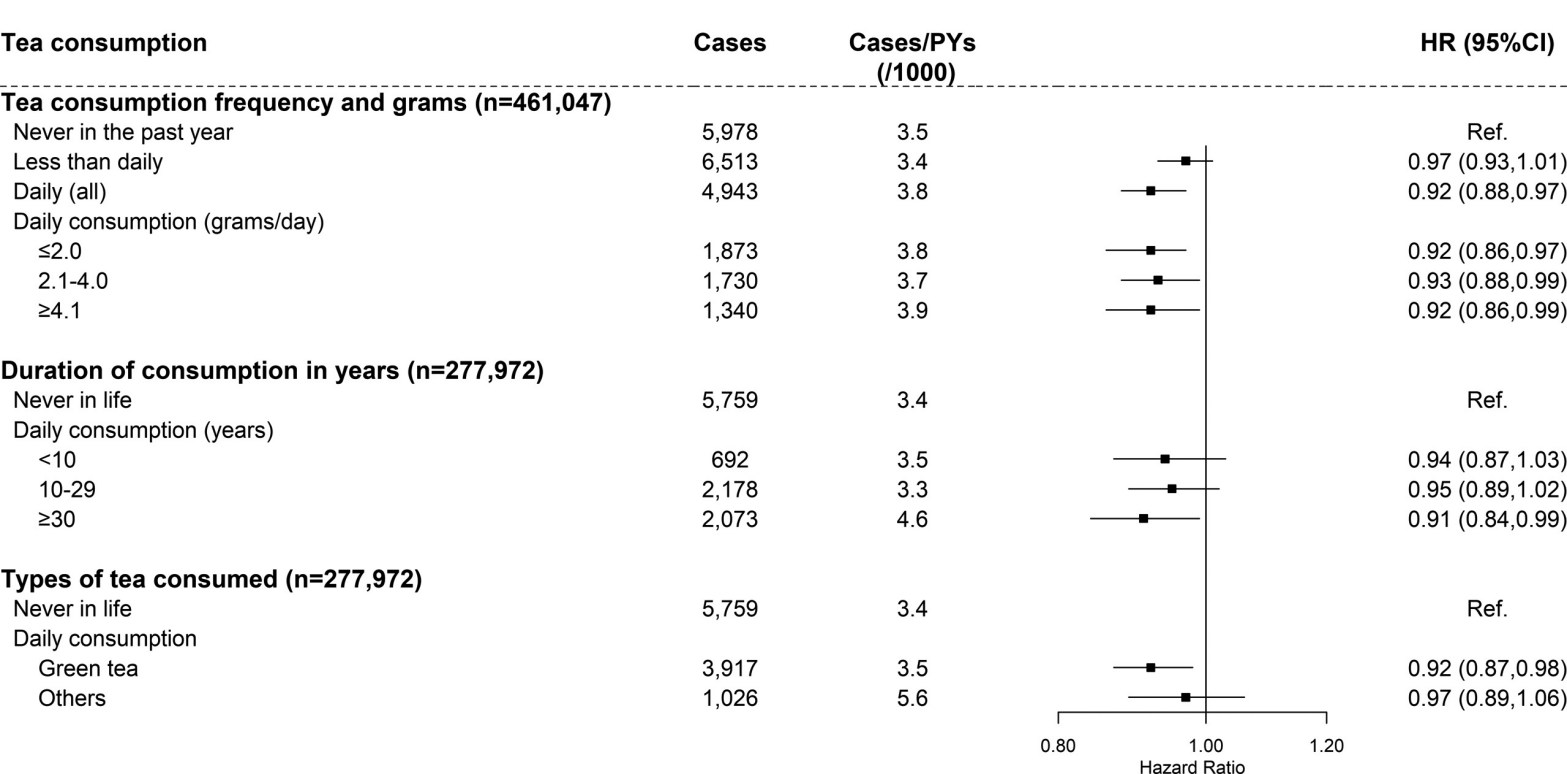
Association between tea consumption and risk of incident type 2 diabetes. Values were obtained from a Cox proportional hazards analysis. Multivariable analyses were adjusted for age (years); education (no formal school, primary school, middle school, high school, college, or university or higher), family history of diabetes (yes or no); smoking (never smoker; former smoker who have quit for reasons other than illness; current smoker or former smoker who has quit because of illness: 1–9, 10–19, 20–29, or ≥30 cigarettes or equivalent/d); alcohol intake (nonweekly drinker; former weekly drinker; weekly drinker; daily drinker: <15, 15–29, 30–59, or ≥60 g/d of pure alcohol); level of physical activity (MET-hours/day); intakes of red meat, fresh vegetables, and fruits (days/week; calculated by assigning participants to the midpoint of their consumption category); BMI (kg/m^2^); waist circumference (centimeters); and prevalent hypertension (yes or no). Solid squares represent the HRs and horizontal lines represent the corresponding 95% CIs. Unadjusted incidence rates are reported per 1000 PYs of follow-up. MET, metabolic equivalent task; PY, person-year; Ref, reference.

During a median of 11.1 y (4,957,160 person-years in total) of follow-up, we identified 17,434 incident T2D cases, with a crude incidence rate of 3.52 cases/1000 person-years. After multivariable adjustment, compared with participants who never consumed tea in the past year, the HRs (95% CIs) for less than daily consumers and daily consumers were 0.97 (0.93, 1.01) and 0.92 (0.88, 0.97) (**Figure 1**), respectively. However, we did not observe a further risk decrease with increasing tea leaves consumed (*P-*trend = 0.999). Compared with participants who never consumed tea, an obvious reduction in risk of T2D was mainly seen in daily consumers who consumed tea for ≥30 y (HR: 0.91; 95% CI: 0.84, 0.99) or who consumed green tea (0.92; 0.87, 0.98).

Sensitivity analyses showed no substantial changes in the results when *1*) excluding T2D cases that occurred during the first 2 follow-up years; *2*) excluding participants with prevalent peptic ulcers at baseline; or *3*) including participants with cancer, stroke, or coronary heart disease at baseline (**Supplemental Table 3**). In subgroup analyses, a stronger association was observed in daily tea consumers who were older, lived in rural areas, or did not have central obesity (defined by WC) (*P-*interaction < 0.05) (**Supplemental Table 4**).

### Tea consumption and risk of all-cause and cause-specific mortality

**FIGURE 2:**
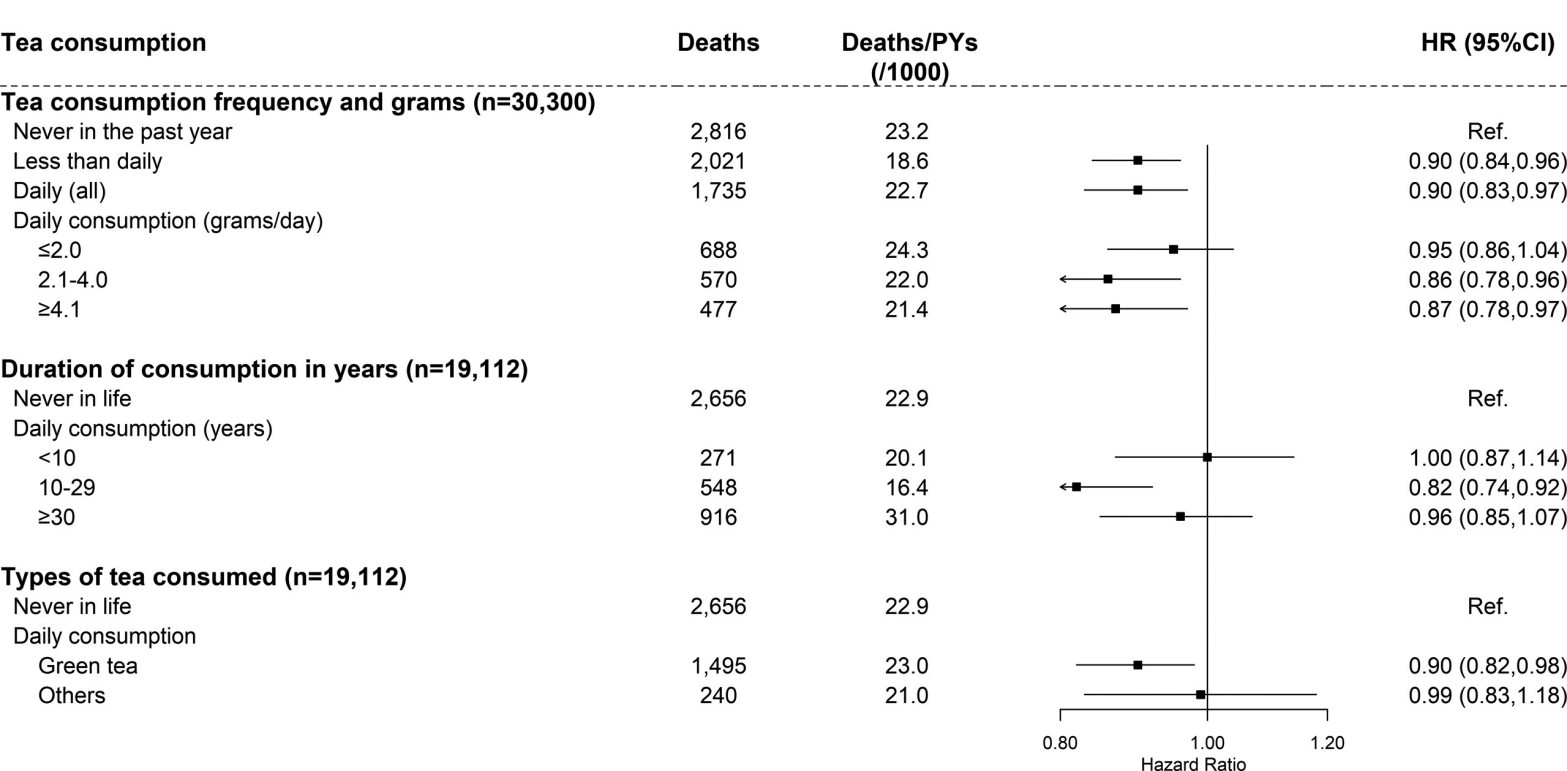
Association between tea consumption and risk of all-cause mortality among diabetic participants. Values were obtained from a Cox proportional hazards analysis. Multivariable analyses were adjusted for age (years); education (no formal school, primary school, middle school, high school, college, or university or higher); random glucose (millimoles/liter); treatment for diabetes (taking insulin and/or oral hypoglycemic drugs, or no treatment); smoking (never smoker; former smoker who has quit for reasons other than illness; current smoker or former smoker who has quit because of illness: 1–9, 10–19, 20–29, or ≥30 cigarettes or equivalent/d); alcohol intake (nonweekly drinker; former weekly drinker; weekly drinker; daily drinker: <15, 15–29, 30–59, or ≥60 g/d of pure alcohol); level of physical activity (MET-hours/day); intakes of red meat, fresh vegetables, and fruits (days/week; calculated by assigning participants to the midpoint of their consumption category); BMI (kg/m^2^); waist circumference (centimeters); and baseline prevalence of hypertension, cancer, stroke, and coronary heart disease (yes or no). Solid squares represent the HRs and horizontal lines represent the corresponding 95% CIs. Unadjusted incidence rates are reported per 1000 PYs of follow-up. MET, metabolic equivalent task; PY, person-year; Ref, reference.

During a median follow-up of 10.6 y (306,194 person-years in total) for 30,300 baseline diabetic participants, 6572 deaths were reported, with a crude mortality of 21.5 deaths/1000 person-years. Compared with participants who never consumed tea in the past year, the multivariable-adjusted HRs (95% CIs) for less than daily consumers and daily consumers were 0.90 (0.84, 0.96) and 0.90 (0.83, 0.97), respectively (**Figure 2**). The mortality risk did not decrease with the increasing amount of tea leaves consumed (*P-*trend = 0.323). Compared with participants who never consumed tea, a reduction in the risk of death was seen in daily consumers who drank tea for 10–29 y (HR: 0.82; 95% CI: 0.74, 0.92) or green tea consumers (0.90; 0.82, 0.98).

The results were not altered when excluding participants with coronary heart disease, stroke, cancer, or chronic kidney disease at baseline from the analysis (**Supplemental Table 5**). The associations were consistent across all subgroups stratified by potential baseline risk factors (*P*-interaction > 0.05) (**Supplemental Table 6**).

Further analyses were performed to examine the association between tea consumption and cause-specific mortality (**Supplemental Table 7**). The mortality risk of cardiovascular diseases, T2D, and others was lower, although to a different extent, for less than daily consumers or daily consumers compared with those who never consumed tea. The HR (95% CI) for cardiovascular death risk of patients with diabetes who consumed tea daily was 0.86 (0.76, 0.96) compared with those who never consumed tea.

### Tea consumption and risk of diabetes complications

**FIGURE 3:**
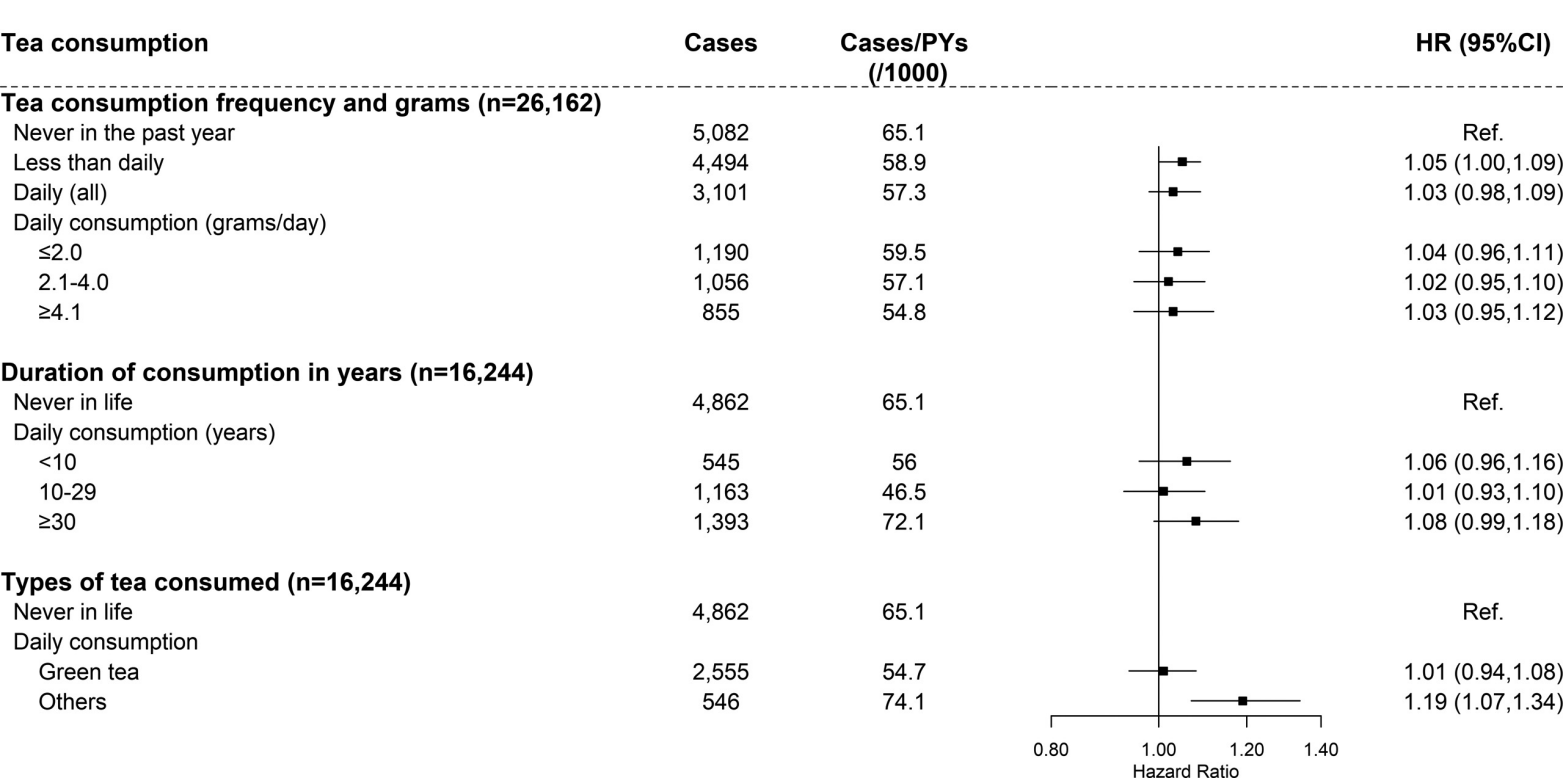
Association between tea consumption and risk of macrovascular complications among diabetic participants without cancer, stroke, or coronary heart disease. Values were obtained from a Cox proportional hazards analysis. Multivariable analyses were adjusted for the same covariates as in Figure 2, except for baseline prevalence of cancer, stroke, and coronary heart disease. Solid squares represent the HRs and horizontal lines represent the corresponding 95% CIs. Unadjusted incidence rates are reported per 1000 PYs of follow-up. PY, person-year; Ref, reference.

**FIGURE 4:**
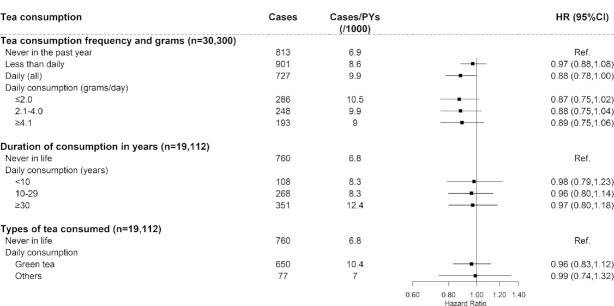
Association between tea consumption and risk of microvascular complications among diabetic participants. Values were obtained from a Cox proportional hazards analysis. Multivariable analyses were adjusted for the same covariates as in Figure 2. Solid squares represent the HRs and horizontal lines represent the corresponding 95% CIs. Unadjusted incidence rates are reported per 1000 PYs of follow-up. PY, person-year; Ref, reference.

Among baseline diabetic participants, we identified 12,677 diabetic macrovascular cases during a median follow-up of 9.6 y (208,384 person-years in total) and 2441 diabetic microvascular cases during a median follow-up of 10.5 y (296,599 person-years in total). Tea consumption was not associated with risk of general or specific diabetic macrovascular complications (**Figure 3**, Supplemental Table 7), except that daily nongreen tea consumers had an increased risk of macrovascular complications (HR: 1.19; 95% CI: 1.07, 1.34). A lower risk was observed for microvascular complications in daily consumers (0.88; 0.78, 1.00) (**Figure 4**) and for diabetic retinopathy in less than daily consumers (0.84; 0.71, 1.00) (Supplemental Table 7). The main results did not change materially in sensitivity analyses (**Supplemental Tables 8** and **9**).

The associations between tea consumption and risk of diabetic complications were consistent across most subgroups stratified by potential baseline risk factors (**Supplemental Tables 10** and **11**), but the magnitude of associations differed for macrovascular complications by alcohol and physical activity and for microvascular complications by prevalent hypertension. For example, the inverse association for microvascular complications was stronger among tea consumers who had prevalent hypertension at baseline than those who did not (*P-*interaction = 0.002) (Supplemental Table 11).

## Discussion

In this population-based cohort of 0.5 million Chinese adults, compared with nonconsumers of tea in the past year, daily green tea consumption was associated with an 8% lower risk of T2D. Among patients with diabetes, daily green tea consumption was associated with a 10% lower risk of death. Daily tea consumption was also associated with a lower risk of diabetic microvascular diseases but not macrovascular complications.

The association between tea consumption and T2D risk is still inconsistent. Our findings are in line with a meta-analysis that included 15 prospective observational studies, although we did not observe a dose–response relation among daily tea consumers, which was found in mainly European cohort studies (10). Altogether, the meta-analysis included 37,445 T2D cases among 545,517 participants over a follow-up period ranging from 3.4 to 24 y, reporting that an increase of 2 cups/d of tea consumption was associated with a 4.6% (95% CI: 0.9%, 8.1%) lower risk of T2D. Studies from the CKB focusing on tea and cardiovascular outcomes also did not find a dose–response relation (29, 30). We speculate that this may be attributed to differences in baseline characteristics between individuals who consumed higher amounts of tea leaves and other consumption groups. For example, those who consumed more tea leaves were more likely to be urban residents, smokers, and have a larger WC. The expected lower risk of T2D in participants who consumed more tea leaves was offset by an increase in other risk factors of T2D. Although we carefully adjusted for those covariates, residual confounding may still mask the dose–response relation.

Another cohort study conducted among Chinese urban residents from the Shanghai Women's Health Study (67,058 females followed up for 10.2 y) and Shanghai Men's Health Study (52,315 males followed up for 5.7 y) reported that current green tea drinkers had an increased risk of T2D compared with noncurrent drinkers (HR: 1.20; 95% CI: 1.14, 1.27) (25). The risk increased with more green tea leaves consumed. The self-reported amount of tea leaves consumed in the Shanghai population (IQR for females: 50–150 g/mo; males: 150–450 g/mo) was much higher than that in our study participants (IQR for females: 28–85 g/mo; males: 57–139 g/mo). However, even the low-consumption group (<100 g/mo for females and <200 g/mo for males) in the Shanghai population showed a higher risk of T2D than nonconsumers. Researchers suggested that the positive association between tea consumption and risk of T2D might be caused by pesticide residue in tea leaves. It has been reported that the use of pesticides coupled with the large surface area of tea leaves makes tea an important source of pesticide exposure to humans (31).

For the association between tea consumption and risk of death and diabetic complications among patients with diabetes, evidence from prospective studies with hard clinical endpoints is rather limited. Our findings that tea consumption was associated with lower diabetes and diabetic complications risks are biologically plausible based on previous studies using surrogate endpoints as the outcome measures. A meta-analysis of 27 randomized controlled trials involving 1898 participants (including patients with diabetes, people at high risk of diabetes, and healthy individuals) (32) showed that green tea consumption was associated with lower concentrations of FBG, which was mainly observed in Asian-based studies. Other meta-analyses of randomized controlled trials among patients with T2D also found that interventions with tea or tea extraction reduced circulating concentrations of C-reactive protein (CRP) (33), maintained a stable fasting blood insulin, and reduced WC (34).

CRP is an important biomarker for predicting cardiovascular risk. The promising findings on green tea and CRP suggested that green tea might reduce the risk of cardiovascular disease by lowering CRP concentrations in T2D patients (33). However, our study did not find significant associations between tea consumption and risk of macrovascular complications in patients with diabetes. We even found elevated risks of diabetic macrovascular complications associated with tea consumption in nongreen tea consumers and some subgroups. One of the possible reasons for these findings is that tea consumers with certain characteristics among baseline patients with diabetes had worse lifestyle habits and glucose control than nonconsumers. Although these factors were carefully controlled in models, there might still be residual confounding. Another finding worth highlighting is that mortality risk decreased in daily tea consumers compared with nonconsumers, especially for the underlying cause of death attributed to cardiovascular diseases. Such inconsistency in findings between incidence and mortality endpoints is worth further investigation.

There have been few cohort studies addressing the risk of diabetic microvascular diseases. Despite certain limitations inherent in the case-control design, 1 hospital-based case-control study in China reported that patients with diabetes who drank green tea every week had a diabetic retinopathy risk reduction of ∼50% compared with those who did not (35). Our study confirmed the potential benefits of tea consumption for diabetic microvascular diseases, especially for diabetic retinopathy.

To the best of our knowledge, this is by far the largest prospective cohort study that comprehensively investigated the association of tea consumption with long-term risk of T2D and risk of death and complications among patients with diabetes. The strengths of our study included the prospective design, the large sample size, the coverage of both urban and rural populations across China, the long-term follow-up, and information on various covariates. We measured tea consumption in grams of tea leaves, which might better reflect the intake of active biochemicals from tea. Our study also extended the analysis by further assessing green tea and other types of tea. Although the causality of the association cannot be established from such an observational study, our findings are statistically robust.

Inevitably, there are some limitations to our study. First, information on tea consumption was self-reported, which might raise the possibility of misclassification. Tea consumption information was collected once at baseline, potentially missing temporal changes occurring during follow-up. Second, there still exists the possibility of residual confounding by other unmeasured or unknown factors, such as total energy intake and treatment for diabetes. Third, a proportion of diabetes cases or diabetic complications cases might not have been detected during follow-up. Most of the cases came from hospitalization records of the health insurance database. However, we linked our participants to local disease registries and conducted an annual survey as a supplement.

In summary, this large cohort study of Chinese adults demonstrated that daily green tea consumption was associated with a long-term lower risk of incident T2D and a lower risk of all-cause mortality in patients with diabetes. However, evidence for other types of tea is still less clear. Importantly, we did not find an increased risk of T2D associated with green tea consumption. The level of pesticides in Chinese tea leaves and its dose–response effect on health need to be confirmed by further studies. Our findings show that there is not sufficient evidence to avoid tea drinking due to concern of pesticides.

## Supplementary Material

nqab006_Supplemental_File

## Data Availability

Data described in the manuscript, code book, and analytic code will be made available from China Kadoorie Biobank upon request (https://www.ckbiobank.org/site/Data±Access).

## Notes

This work was supported by the National Natural Science Foundation of China (81941018). The China Kadoorie Biobank baseline survey and the first re-survey were supported by a grant from the Kadoorie Charitable Foundation in Hong Kong. The long-term follow-up is supported by grants 2016YFC0900500, 2016YFC0900501 and 2016YFC0900504 from the National Key R&D Program of China, the National Natural Science Foundation of China (81390540, 81390541, 81390544), and the Chinese Ministry of Science and Technology (2011BAI09B01).

The funders had no role in the study design, data collection, data analysis and interpretation, writing of the report, or the decision to submit the article for publication.

Supplemental Figure 1 and Supplemental Tables 1–11 are available from the “Supplementary data” link in the online posting of the article and from the same link in the online table of contents at https://academic.oup.com/ajcn/.

Abbreviations used: CKB, China Kadoorie Biobank; CRP, C-reactive protein; FBG, fasting blood glucose; HI, National Health Insurance; ICD-10, International Classification of Diseases, 10th revision; MET, metabolic equivalent task; T2D, type 2 diabetes; WC, waist circumference.
