# Supplementary material for: Tea consumption and long-term risk of type 2 diabetes and diabetic complications: a cohort study of 0.5 million Chinese adults
Source: Am J Clin Nutr. 2021 Mar 11;114(1):194–202. doi: 10.1093/ajcn/nqab006 (PMC8246622; doi:10.1093/ajcn/nqab006)
Supplement: nqab006_Supplemental_File [file nqab006_supplemental_file.docx]

On-line Supplementary Material

Tea consumption and long-term risk of type 2 diabetes and diabetic complications: A cohort study of 0.5 million Chinese adults

First author: Jia Nie

# **Supplementary figure 1. Flow chart of participants inclusion process.**


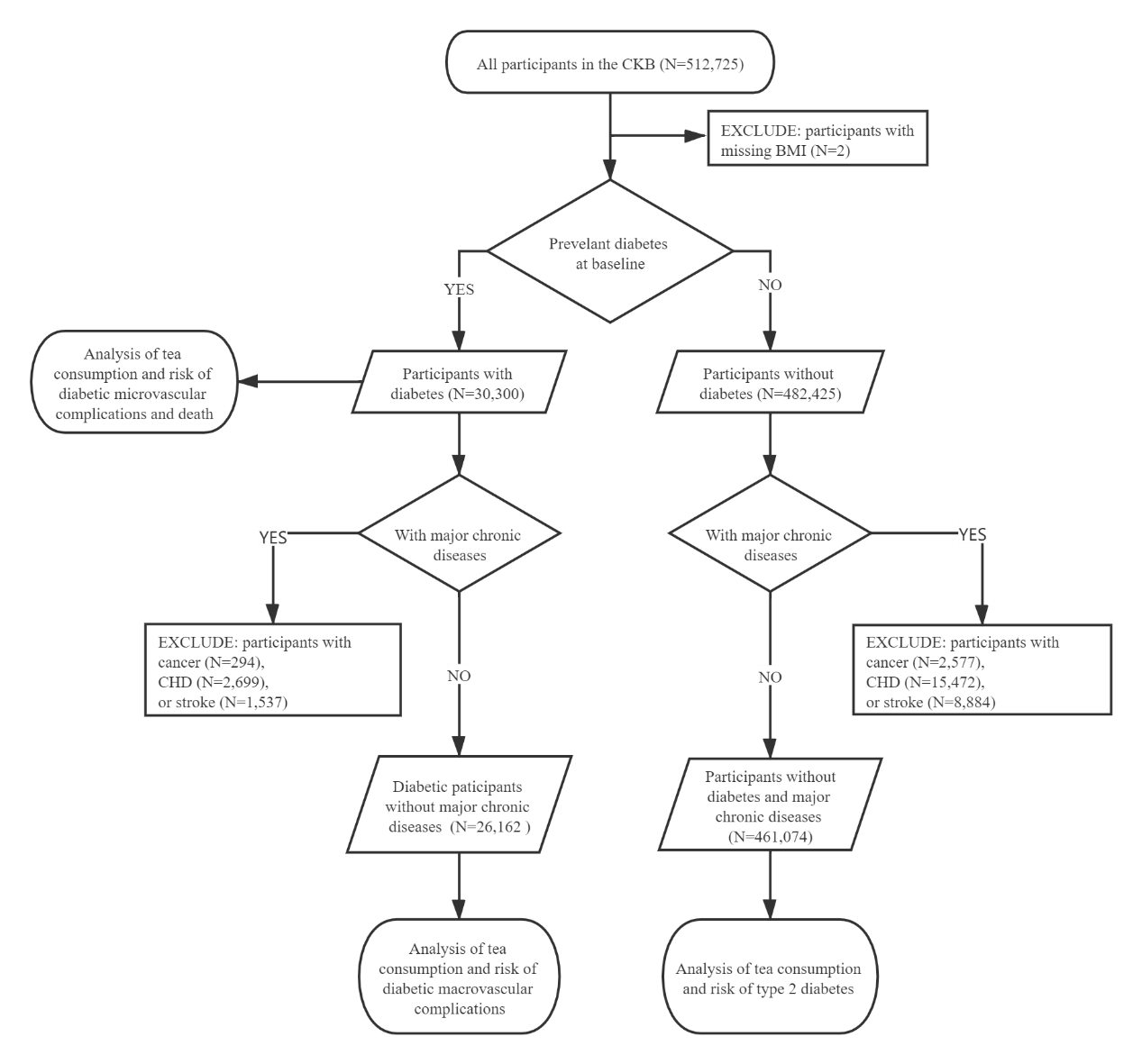


# **Supplementary Table 1. ICD-10 codes for study outcomes.**

| **Outcomes** | **ICD-10 codes** |
| --- | --- |
| Type 2 diabetes | E11, E14 |
| Cardiovascular diseases | I00-I25, I27-I88, I95-I99 |
| Diabetes complications |  |
| Macrovascular complications |  |
| Ischemic heart disease | I20-I25 |
| Stroke | I60, I61, I63, I64 |
| Other macrovascular diseases | I00-I19, I27-I59, I62, I65-I88, I95-I99, E10.5, E11.5, E12.5, E13.5, E14.5 |
| Microvascular complications |  |
| Diabetic retinopathy | E10.3, E11.3, E12.3, E13.3, E14.3, H36.0 |
| Diabetic nephropathy | E10.2, E11.2, E12.2, E13.2, E14.2 |
| Diabetic neuropathy | E10.4, E11.4, E12.4, E13.4, E14.4 |

# **Supplementary Table 2. Baseline characteristics of participants with diabetes according to tea consumption**

|  | N=30,300 ^a^ | | |  | N=26,162^b^ | | |
| --- | --- | --- | --- | --- | --- | --- | --- |
|  | Never in the past year | Less than daily | Daily |  | Never in the past year | Less than daily | Daily |
| No. of participants (%) | 12,070 (39.8) | 10,639 (35.1) | 7,591 (25.1) |  | 10,055 (38.4) | 9,404 (36.0) | 6,703 (25.6) |
| Female, % | 77.6 | 59.1 | 39.0 |  | 78.8 | 59.0 | 38.0 |
| Age (S.D), year | 59.4 (9.9) | 57.0 (9.3) | 58.0 (9.6) |  | 58.5 (10.0) | 56.2 (9.7) | 57.3 (9.8) |
| Urban, % | 59.0 | 63.1 | 60.5 |  | 55.8 | 60.9 | 58.9 |
| Middle school or higher, % | 41.8 | 48.9 | 50.5 |  | 40.9 | 48.0 | 49.4 |
| Current smoker ^c^, % |  |  |  |  |  |  |  |
| Male | 54.7 | 58.3 | 70.3 |  | 54.3 | 58.7 | 70.8 |
| Female | 3.7 | 4.3 | 7.1 |  | 3.2 | 4.0 | 6.7 |
| Weekly or daily alcohol drinker, % |  |  |  |  |  |  |  |
| Male | 21.5 | 27.9 | 37.2 |  | 22.9 | 29.5 | 39.1 |
| Female | 0.7 | 1.8 | 3.7 |  | 0.7 | 1.7 | 3.5 |
| Physical activity (S.D), MET-h/d | 15.2 (10.9) | 15.8 (10.2) | 15.1 (11.1) |  | 16.2 (11.2) | 16.6 (10.5) | 15.8 (11.5) |
| Regular consumption ≥ 4 days/week, % |  |  |  |  |  |  |  |
| Red meat | 48.7 | 52.7 | 55.1 |  | 49.1 | 52.4 | 54.9 |
| Fresh vegetables | 98.7 | 98.5 | 99.0 |  | 98.6 | 98.4 | 99.0 |
| Fresh fruits | 25.0 | 30.2 | 34.8 |  | 23.9 | 28.8 | 33.4 |
| Physical measurements |  |  |  |  |  |  |  |
| BMI (S.D), kg/m^2^ | 24.8 (3.7) | 25.2 (3.5) | 25.4 (3.7) |  | 24.7 (3.7) | 25.0 (3.5) | 25.2 (3.8) |
| WC (S.D), mm | 851.2 (102.4) | 861.7 (95.7) | 866.7 (104.1) |  | 846.1 (103.0) | 857.4 (95.9) | 862.2 (104.3) |
| Postmenopausal women, % | 79.9 | 79.6 | 79.9 |  | 77.6 | 77.3 | 77.7 |
| Family medical history, % |  |  |  |  |  |  |  |
| Heart attack | 3.7 | 3.7 | 4.8 |  | 3.5 | 3.4 | 4.4 |
| Stroke | 20.5 | 22.7 | 21.7 |  | 19.2 | 21.3 | 20.3 |
| Cancer | 18.1 | 18.9 | 19.5 |  | 17.6 | 18.4 | 18.7 |
| Baseline prevalent diseases, % |  |  |  |  |  |  |  |
| Hypertension | 58.7 | 60.4 | 62.0 |  | 55.9 | 58.3 | 59.6 |
| Coronary heart disease | 9.3 | 8.4 | 8.8 |  | - | - | - |
| Stroke | 6.0 | 4.4 | 4.1 |  | - | - | - |
| Cancer | 1.1 | 1.0 | 0.8 |  | - | - | - |
| Random glucose (S.D), mmol/L | 11.9 (5.9) | 12.1 (5.5) | 12.8 (6.0) |  | 12.0 (5.9) | 12.2 (5.5) | 12.9 (6.0) |
| Diabetic treatment ^d^, % | 35.0 | 35.2 | 33.5 |  | 32.3 | 33.0 | 31.0 |
| Years since diagnosis of diabetes (S.D), year | 6.3 (7.4) | 6.1 (7.2) | 6.2 (7.9) |  | 5.9 (7.3) | 5.9 (7.1) | 5.9 (7.9) |
| Tea consumption |  |  |  |  |  |  |  |
| Consumption years (S.D), year | - | - | 25.9 (11.6) |  | - | - | 25.5 (11.3) |
| Green tea consumption, % | - | - | 85.4 |  | - | - | 84.6 |

Abbreviations: BMI: body mass index; MET, metabolic equivalent of task; S.D, standard deviation; WC: waist circumference.

Values are means or percentages and were adjusted for age, sex, and region, where appropriate, using either multiple linear regression (for continuous outcomes) or logistic regression (for binary outcomes).

^a^ Including all participants with diabetes at baseline.

^b^ Including participants with diabetes at baseline but excluding those with cancer, coronary heart diseases, and stroke.

^c^ Former smoker who had stopped smoking for illness was categorized into the current smoker.

^d^ Diabetic treatment includes insulin and/or oral hypoglycemic drugs.

# **Supplementary Table 3. Sensitivity analyses of the association between tea consumption and risk of T2D.**

|  | Never in the past year | Less than daily | Daily (all) | Daily consumption (grams/day) | | |
| --- | --- | --- | --- | --- | --- | --- |
|  |  |  |  | ≤2.0 | 2.1-4.0 | ≥4.1 |
| **Excluding cases identified during the first 2 years of follow-up** | | | | | | |
| Cases | 5,367 | 6,061 | 4,613 | 1,751 | 1,626 | 1,236 |
| Person years | 1720212 | 1936500 | 1298796 | 496305.8 | 462178.3 | 340311.6 |
| Cases/PYs (/1000) | 3.12 | 3.13 | 3.55 | 3.53 | 3.52 | 3.63 |
| HR (95%CI) | 1.00 | 0.98 (0.94,1.02) | 0.93 (0.88,0.98) | 0.92 (0.87,0.98) | 0.94 (0.88,1.01) | 0.92 (0.86,0.99) |
| **Excluding participants with prevalent peptic ulcers at baseline** | | | | | | |
| Cases | 5,751 | 6,231 | 4,762 | 1,813 | 1,670 | 1,279 |
| Person years | 1652245 | 1858785 | 1254904 | 480271.9 | 448354.9 | 326277.1 |
| Cases/PYs (/1000) | 3.48 | 3.35 | 3.79 | 3.77 | 3.72 | 3.92 |
| HR (95%CI) | 1.00 | 0.97 (0.93,1.00) | 0.92 (0.88,0.97) | 0.92 (0.86,0.97) | 0.93 (0.87,0.99) | 0.92 (0.86,0.98) |
| **Including those who had prevalent cancer, stroke or coronary heart diseases at baseline** | | | | | | |
| Cases | 6,484 | 6,940 | 5,266 | 2,008 | 1,831 | 1,427 |
| Person years | 1816261 | 2003580 | 1344731 | 514335.1 | 478459.6 | 351936.5 |
| Cases/PYs (/1000) | 3.57 | 3.46 | 3.92 | 3.90 | 3.83 | 4.05 |
| HR (95%CI) | 1.00 | 0.98 (0.94,1.01) | 0.94 (0.89,0.98) | 0.94 (0.88,0.99) | 0.94 (0.88,1.00) | 0.94 (0.88,1.00) |

Abbreviations: CI, confidence interval; HR, hazard ratio; PYs, person years.

Values were obtained from Cox proportional hazards analysis. Models were adjusted for the same variables in Figure 1, as appropriate.

# **Supplementary Table 4. Subgroup analysis of associations between tea consumption and risk of type 2 diabetes according to potential baseline risk factors (n=461,047).**

|  | Never in the past year | |  | Less than daily | |  | Daily | | *P_i_*_nteraction_ |
| --- | --- | --- | --- | --- | --- | --- | --- | --- | --- |
|  | Cases | HR |  | Cases | HR (95% CI) |  | Cases | HR (95% CI) |  |
| Age at baseline (years) |  |  |  |  |  |  |  |  | <0.001 |
| <50 | 1,637 | 1.00 |  | 2,483 | 1.05 (0.98,1.12) |  | 1,674 | 0.98 (0.90,1.07) |  |
| 50-59 | 2,326 | 1.00 |  | 2,369 | 0.94 (0.88,1.00) |  | 1,828 | 0.90 (0.83,0.97) |  |
| ≥60 | 2,015 | 1.00 |  | 1,661 | 0.96 (0.89,1.03) |  | 1,441 | 0.91 (0.83,0.99) |  |
| Sex |  |  |  |  |  |  |  |  | 0.930 |
| Female | 4,879 | 1.00 |  | 4,016 | 0.98 (0.93,1.02) |  | 1,813 | 0.93 (0.87,1.00) |  |
| Male | 1,099 | 1.00 |  | 2,497 | 0.96 (0.89,1.04) |  | 3,130 | 0.92 (0.85,0.99) |  |
| Region |  |  |  |  |  |  |  |  | 0.024 |
| Urban | 2,571 | 1.00 |  | 2,761 | 1.00 ( 0.95,1.07) |  | 1,888 | 0.99 (0.92,1.07) |  |
| Rural | 3,407 | 1.00 |  | 3,752 | 0.94 (0.89,0.99) |  | 3,055 | 0.88 (0.82,0.93) |  |
| Education |  |  |  |  |  |  |  |  | 0.081 |
| Middle school or higher | 1,725 | 1.00 |  | 2,891 | 1.02 (0.95,1.08) |  | 2,118 | 0.97 (0.90,1.04) |  |
| Primary school or lower | 4,253 | 1.00 |  | 3,622 | 0.95 (0.90,1.00) |  | 2,825 | 0.91 (0.86,0.97) |  |
| Smoking status |  |  |  |  |  |  |  |  | 0.468 |
| Current ^a^ | 743 | 1.00 |  | 1,845 | 1.00 (0.92,1.10) |  | 2,436 | 0.95 (0.86,1.04) |  |
| Not current | 5,235 | 1.00 |  | 4,668 | 0.96 (0.92,1.01) |  | 2,507 | 0.93 (0.88,0.99) |  |
| Alcohol consumption |  |  |  |  |  |  |  |  | 0.789 |
| Weekly or daily | 344 | 1.00 |  | 944 | 0.99 (0.87,1.13) |  | 1,296 | 0.98 (0.86,1.11) |  |
| Less than weekly | 5,634 | 1.00 |  | 5,569 | 0.97 (0.93,1.01) |  | 3,647 | 0.92 (0.87,0.97) |  |
| Fresh fruit consumption |  |  |  |  |  |  |  |  | 0.916 |
| Daily | 1,002 | 1.00 |  | 1,213 | 0.95 (0.87,1.04) |  | 830 | 0.91 (0.82,1.02) |  |
| Less than daily | 4,976 | 1.00 |  | 5,300 | 0.97 (0.93,1.02) |  | 4,113 | 0.93 (0.88,0.98) |  |
| Physical activity (MET h/day) |  |  |  |  |  |  |  |  | 0.522 |
| Male<10.79/Female<11.20 | 1,634 | 1.00 |  | 1,639 | 1.02 (0.95,1.10) |  | 1,457 | 0.94 (0.86,1.03) |  |
| Male10.79-19.97/Female11.20-17.69 | 1,421 | 1.00 |  | 1,693 | 0.94 (0.87,1.02) |  | 1,419 | 0.92 (0.84,1.01) |  |
| Male19.98-33.32/Female17.70-29.14 | 1,446 | 1.00 |  | 1,686 | 0.94 (0.87,1.01) |  | 1,095 | 0.93 (0.85,1.03) |  |
| Male≥33.33/Female≥29.15 | 1,477 | 1.00 |  | 1,495 | 0.98 (0.91,1.06) |  | 972 | 0.92 (0.83,1.01) |  |
| BMI (kg/m^2^) |  |  |  |  |  |  |  |  | 0.011 |
| ≥24.0 | 3,911 | 1.00 |  | 4,410 | 0.97 (0.93,1.02) |  | 3,296 | 0.93 (0.88,0.99) |  |
| <24.0 | 2,067 | 1.00 |  | 2,103 | 0.98 (0.91,1.05) |  | 1,647 | 0.93 (0.85,1.01) |  |
| WC (cm) |  |  |  |  |  |  |  |  | <0.001 |
| Male≥85/Female≥80 | 3,759 | 1.00 |  | 4,266 | 0.98 (0.93,1.03) |  | 3,228 | 0.95 (0.90,1.01) |  |
| Male<85/Female<80 | 2,219 | 1.00 |  | 2,247 | 0.95 (0.89,1.01) |  | 1,715 | 0.88 (0.82,0.96) |  |
| Prevalent hypertension |  |  |  |  |  |  |  |  | 0.684 |
| Yes | 3,296 | 1.00 |  | 3,091 | 0.96 (0.91,1.02) |  | 2,662 | 0.94 (0.88,1.01) |  |
| No | 2,682 | 1.00 |  | 3,422 | 0.97 (0.92,1.03) |  | 2,281 | 0.90 (0.84,0.97) |  |
| Family history of diabetes |  |  |  |  |  |  |  |  | 0.119 |
| Yes | 549 | 1.00 |  | 777 | 1.05 (0.93,1.18) |  | 472 | 0.93 (0.80,1.08) |  |
| No | 5,429 | 1.00 |  | 5,736 | 0.96 (0.92,1.00) |  | 4,471 | 0.92 (0.88,0.97) |  |

Abbreviations: HR, hazard ratio; CI, confidence interval; MET, metabolic equivalent of task (grouped by quartailes acording to sex); BMI: body mass index; WC: waist circumference.

Values were obtained from Cox proportional hazards analysis. Except for the baseline stratifying variable, the model was adjusted for the same covariates as in the model of Figure 1. *P*_interaction_, *P* from the likelihood ratio tests for interaction.

^a^ Former smoker who had stopped smoking for illness was categorized into the current smoker.

# **Supplementary Table 5. Sensitivity analyses of the association between tea consumption and risk of all-cause mortality in diabetic patients.**

|  | Never in the past year | Less than daily | Daily (all) | Daily consumption (grams/day) | | |
| --- | --- | --- | --- | --- | --- | --- |
|  |  |  |  | ≤2.0 | 2.1-4.0 | ≥4.1 |
| **Excluding thoese who had prevalent cancer, stroke or coronary heart diseases at baseline** | | | | | | |
| Cases | 2,067 | 1,616 | 1,424 | 570 | 460 | 394 |
| Person years | 102597.7 | 96873.4 | 68039.8 | 25156.8 | 23235.4 | 19647.6 |
| Cases/PYs (/1000) | 20.15 | 16.68 | 20.93 | 22.66 | 19.80 | 20.05 |
| HR (95%CI) | 1.00 | 0.89 (0.83,0.96) | 0.91 (0.84,0.99) | 0.95 (0.85,1.05) | 0.86 (0.76,0.96) | 0.93 (0.82,1.05) |
| **Excluding thoese who had prevalent cancer, stroke, coronary heart diseases or chronic kidney diseases at baseline** | | | | | | |
| Cases | 2,026 | 1,574 | 1,398 | 560 | 452 | 386 |
| Person years | 100578.5 | 95018.5 | 67215.4 | 24851.1 | 22976.4 | 19388.0 |
| Cases/PYs (/1000) | 20.14 | 16.57 | 20.80 | 22.53 | 19.67 | 19.91 |
| HR (95%CI) | 1.00 | 0.89 (0.82,0.95) | 0.91 (0.84,0.99) | 0.94 (0.85,1.05) | 0.86 (0.77,0.97) | 0.93 (0.82,1.05) |

Abbreviations: CI, confidence interval; HR, hazard ratio; PYs, person years.

Values were obtained from Cox proportional hazards analysis. Models were adjusted for the same variables in Figure 2, as appropriate.

# **Supplementary Table 6. Subgroup analysis of associations between tea consumption and risk of all-cause mortality according to potential baseline risk factors (n=30,300).**

| Subgroups | Never in the past year | |  | Less than daily | |  | Daily | | *P_i_*_nteraction_ |
| --- | --- | --- | --- | --- | --- | --- | --- | --- | --- |
|  | Death | HR |  | Death | HR (95% CI) |  | Death | HR (95% CI) |  |
| Age at baseline (years) |  |  |  |  |  |  |  |  | 0.349 |
| <50 | 157 | 1.00 |  | 204 | 1.02 (0.81,1.28) |  | 124 | 1.00 (0.75,1.33) |  |
| 50-59 | 584 | 1.00 |  | 513 | 0.95 (0.83,1.08) |  | 432 | 1.02 (0.87,1.19) |  |
| ≥60 | 2,075 | 1.00 |  | 1,304 | 0.88 (0.82,0.95) |  | 1,179 | 0.85 (0.78,0.93) |  |
| Sex |  |  |  |  |  |  |  |  | 0.074 |
| Female | 1,936 | 1.00 |  | 1,020 | 0.90 (0.83,0.98) |  | 600 | 1.01 (0.90,1.13) |  |
| Male | 880 | 1.00 |  | 1,001 | 0.87 (0.79,0.96) |  | 1,135 | 0.83 (0.75,0.92) |  |
| Region |  |  |  |  |  |  |  |  | 0.795 |
| Urban | 1,577 | 1.00 |  | 1,136 | 0.91 (0.83,0.98) |  | 875 | 0.88 (0.80,0.97) |  |
| Rural | 1,239 | 1.00 |  | 885 | 0.89 (0.81,0.98) |  | 860 | 0.91 (0.81,1.02) |  |
| Education |  |  |  |  |  |  |  |  | 0.931 |
| Middle school or higher | 852 | 1.00 |  | 817 | 0.88 (0.79,0.97) |  | 678 | 0.87 (0.77,0.98) |  |
| Primary school or lower | 1,964 | 1.00 |  | 1,204 | 0.89 (0.82,0.97) |  | 1,057 | 0.88 (0.80,0.97) |  |
| Smoking status |  |  |  |  |  |  |  |  | 0.398 |
| Current ^a^ | 623 | 1.00 |  | 707 | 0.90 (0.80,1.01) |  | 883 | 0.87 (0.77,0.98) |  |
| Not current | 2,193 | 1.00 |  | 1,314 | 0.90 (0.84,0.97) |  | 852 | 0.93 (0.84,1.02) |  |
| Alcohol consumption |  |  |  |  |  |  |  |  | 0.275 |
| Weekly or daily | 156 | 1.00 |  | 251 | 0.82 (0.66,1.01) |  | 342 | 0.75 (0.60,0.93) |  |
| Less than weekly | 2,660 | 1.00 |  | 1,770 | 0.90 (0.84,0.96) |  | 1,393 | 0.93 (0.85,1.00) |  |
| Fresh fruit consumption |  |  |  |  |  |  |  |  | 0.113 |
| ≥ 4 days/week | 601 | 1.00 |  | 495 | 0.91 (0.80,1.03) |  | 449 | 0.97 (0.83,1.12) |  |
| < 4 days/week | 2,215 | 1.00 |  | 1,526 | 0.89 (0.83,0.96) |  | 1,286 | 0.85 (0.78,0.93) |  |
| Physical activity (MET h/day) | |  |  |  |  |  |  |  | 0.133 |
| Male<5.65/Female<8.40 | 1,074 | 1.00 |  | 665 | 0.91 (0.82,1.02) |  | 599 | 0.93 (0.82,1.06) |  |
| Male5.65-12.19/Female8.40-11.69 | 867 | 1.00 |  | 594 | 0.84 (0.74,0.94) |  | 540 | 0.80 (0.69,0.92) |  |
| Male12.20-23.44/Female11.70-18.19 | 548 | 1.00 |  | 424 | 0.82 (0.71,0.95) |  | 355 | 0.82 (0.69,0.97) |  |
| Male≥23.45/Female≥18.20 | 327 | 1.00 |  | 338 | 1.09 (0.92,1.29) |  | 241 | 1.15 (0.93,1.42) |  |
| BMI (kg/m^2^) |  |  |  |  |  |  |  |  | 0.931 |
| ≥24.0 | 1,544 | 1.00 |  | 1,132 | 0.92 (0.85,1.00) |  | 919 | 0.92 (0.83,1.02) |  |
| <24.0 | 1,272 | 1.00 |  | 889 | 0.86 (0.78,0.95) |  | 816 | 0.88 (0.78,0.98) |  |
| WC (cm) |  |  |  |  |  |  |  |  | 0.390 |
| Male≥85/Female≥80 | 1,838 | 1.00 |  | 1,307 | 0.88 (0.82,0.95) |  | 1,058 | 0.87 (0.80,0.96) |  |
| Male<85/Female<80 | 978 | 1.00 |  | 714 | 0.92 (0.83,1.03) |  | 677 | 0.96 (0.84,1.09) |  |
| Prevalent hypertension |  |  |  |  |  |  |  |  | 0.494 |
| Yes | 2,057 | 1.00 |  | 1,396 | 0.89 (0.83,0.96) |  | 1,233 | 0.89 (0.81,0.97) |  |
| No | 759 | 1.00 |  | 625 | 0.90 (0.80,1.01) |  | 502 | 0.91 (0.79,1.05) |  |
| Diabetic treatment ^b^ |  |  |  |  |  |  |  |  | 0.993 |
| Yes | 1,170 | 1.00 |  | 851 | 0.90 (0.82,0.99) |  | 672 | 0.97 (0.86,1.09) |  |
| No | 1,646 | 1.00 |  | 1,170 | 0.90 (0.83,0.98) |  | 1,063 | 0.91 (0.83,1.01) |  |

Abbreviations: HR, hazard ratio; CI, confidence interval; MET, metabolic equivalent of task (grouped by quartailes acording to sex); BMI: body mass index; WC: waist circumference.

Values were obtained from Cox proportional hazards analysis. Except for the baseline stratifying variable, the model was adjusted for the same variables as in the model of Figure 2. *P*_interaction_, *P* from the likelihood ratio tests for interaction.

^a^ Former smoker who had stopped smoking for illness was categorized into the current smoker.

^b^ Diabetic treatment includes insulin and/or oral hypoglycemic drugs.

# **Supplementary Table 7. Association of tea consumption with the risk of cause-specific mortality and diabetic complications among diabetic participants.**

| Tea consumption | Causes-specific mortality ^a^ | | |  | Macrovascular complications ^b^ | | |  | Microvascular complications ^a^ | | |
| --- | --- | --- | --- | --- | --- | --- | --- | --- | --- | --- | --- |
|  | (n=30,300) | | |  | (n=26,162) | | |  | (n=30,300) | | |
|  | Death | Death/PYs (/1000) | HR (95%CI) |  | Cases | Cases/PYs (/1000) | HR (95%CI) |  | Cases | Cases/PYs (/1000) | HR (95%CI) |
|  | Cardiovascular diseases ^c^ | | |  | Ischemic heart disease ^d^ | | |  | Diabetic nephropathy ^f^ | | |
| Never in the past year | 1,318 | 10.88 | 1.00 |  | 1,964 | 20.67 | 1.00 |  | 304 | 2.59 | 1.00 |
| Less than daily | 905 | 8.34 | 0.94 (0.86,1.03) |  | 1,708 | 18.95 | 1.04 (0.97,1.12) |  | 335 | 3.18 | 1.07 (0.91,1.27) |
| Daily | 704 | 9.20 | 0.86 (0.76,0.96) |  | 1,223 | 19.30 | 1.06 (0.97,1.16) |  | 264 | 3.53 | 0.99 (0.81,1.21) |
|  | T2D | | |  | Stroke ^e^ | | |  | Diabetic retinopathy | | |
| Never in the past year | 396 | 3.27 | 1.00 |  | 2,403 | 25.81 | 1.00 |  | 334 | 2.80 | 1.00 |
| Less than daily | 293 | 2.70 | 0.82 (0.70,0.98) |  | 2,016 | 22.61 | 1.01 (0.94,1.07) |  | 309 | 2.88 | 0.84 (0.71,1.00) |
| Daily | 278 | 3.63 | 0.95 (0.78,1.16) |  | 1,340 | 21.22 | 0.96 (0.88,1.04) |  | 285 | 3.79 | 0.88 (0.73,1.07) |
|  | Others | | |  | Other macrovascular diseases | | |  | Diabetic neuropathy | | |
| Never in the past year | 1,102 | 9.10 | 1.00 |  | 3,352 | 38.53 | 1.00 |  | 311 | 2.60 | 1.00 |
| Less than daily | 823 | 7.58 | 0.86 (0.78,0.95) |  | 3,017 | 35.87 | 1.04 (0.99,1.10) |  | 410 | 3.84 | 1.00 (0.85,1.17) |
| Daily | 753 | 9.85 | 0.91 (0.81,1.02) |  | 2,132 | 36.07 | 1.04 (0.97,1.12) |  | 314 | 4.18 | 0.89 (0.74,1.08) |

Abbreviations: CI, confidence interval; HR, hazard ratio; PYs, person years.

Values were obtained from Cox proportional hazards analysis.

^a^ Adjusted for the same covariates as in Figure 2.

^b^ Adjusted for the same covariates as in Figure 2, except for baseline prevalence of cancer, stroke, and coronary heart disease.

^c^ Additionally adjusted for family history of heart attack and stroke.

^d^ Additionally adjusted for family history of heart attack.

^e^ Additionally adjusted for family history of stroke.

^f^ The analyses further excluded participants who reported having chronic kidney disease at baseline.

# **Supplementary Table 8. Sensitivity analyses of the association between tea consumption and risk macrovascular complications in diabetic patients without cancer, stroke or coronary heart diseases.**

|  | Never in the past year | Less than daily | Daily (all) | Daily consumption (grams/day) | | |
| --- | --- | --- | --- | --- | --- | --- |
|  |  |  |  | ≤2.0 | 2.1-4.0 | ≥4.1 |
| **Excluding cases identified during the first 2 years of follow-up** | | |  |  |  |  |
| Cases | 4,001 | 3,693 | 2,522 | 971 | 870 | 681 |
| Person years | 76983.9 | 75435.6 | 53479.9 | 19765.2 | 18299.5 | 15415.2 |
| Cases/PYs (/1000) | 52.0 | 49.0 | 47.2 | 49.1 | 47.5 | 44.2 |
| HR (95%CI) | 1.00 | 1.06 (1.01,1.12) | 1.05 (0.99,1.12) | 1.05 (0.97,1.14) | 1.05 (0.97,1.14) | 1.05 (0.96,1.15) |
| **Excluding thoese who had prevalent chronic kidney diseases at baseline** | | | | | | |
| Cases | 3,908 | 3,610 | 2,486 | 962 | 855 | 669 |
| Person years | 75555.3 | 74077.6 | 52936.2 | 19572.4 | 18130.5 | 15233.2 |
| Cases/PYs (/1000) | 51.7 | 48.7 | 47.0 | 49.2 | 47.2 | 43.9 |
| HR (95%CI) | 1.00 | 1.06 (1.01,1.12) | 1.05 (0.99,1.12) | 1.05 (0.97,1.14) | 1.05 (0.96,1.14) | 1.05 (0.95,1.15) |

Abbreviations: CI, confidence interval; HR, hazard ratio; PYs, person years.

Values were obtained from Cox proportional hazards analysis. Models were adjusted for the same variables in Figure 3, as appropriate.

# **Supplementary Table 9. Sensitivity analyses of the association between tea consumption and risk of microvascular complications in diabetic patients.**

|  | Never in the past year | Less than daily | Daily (all) | Daily consumption (grams/day) | | |
| --- | --- | --- | --- | --- | --- | --- |
|  |  |  |  | ≤2.0 | 2.1-4.0 | ≥4.1 |
| **Excluding cases identified during the first 2 years of follow-up** | | | | | | |
| Cases | 676 | 804 | 653 | 263 | 222 | 168 |
| Person years | 117596.90 | 105054.3 | 73597.92 | 27203.31 | 24930.53 | 21464.08 |
| Cases/PYs (/1000) | 5.75 | 7.65 | 8.87 | 9.67 | 8.90 | 7.83 |
| HR (95%CI) | 1.00 | 1.01 (0.90,1.12) | 0.90 (0.79,1.03) | 0.90 (0.77,1.06) | 0.90 (0.76,1.07) | 0.89 (0.74,1.08) |
| **Excluding thoese who had prevalent cancer, stroke or coronary heart diseases at baseline** | | | | | | |
| Cases | 653 | 795 | 628 | 255 | 213 | 160 |
| Person years | 99810.8 | 93888.5 | 65659.4 | 24247.5 | 22453.8 | 18958.1 |
| Cases/PYs (/1000) | 6.54 | 8.47 | 9.56 | 10.52 | 9.49 | 8.44 |
| HR (95%CI) | 1.00 | 0.99 (0.88,1.11) | 0.87 (0.76,0.99) | 0.88 (0.75,1.04) | 0.84 (0.71,1.00) | 0.88 (0.72,1.06) |
| **Excluding thoese who had prevalent cancer, stroke, coronary heart diseases or chronic kidney diseases at baseline** | | | | | | |
| Cases | 638 | 773 | 622 | 253 | 211 | 158 |
| Person years | 97836.2 | 92113.8 | 64863.3 | 23955.8 | 22197.5 | 18709.9 |
| Cases/PYs (/1000) | 6.52 | 8.39 | 9.59 | 10.56 | 9.51 | 8.44 |
| HR (95%CI) | 1.00 | 0.99 (0.88,1.11) | 0.88 (0.77,1.01) | 0.89 (0.76,1.06) | 0.85 (0.71,1.01) | 0.89 (0.73,1.09) |

Abbreviations: CI, confidence interval; HR, hazard ratio; PYs, person years.

Values were obtained from Cox proportional hazards analysis. Models were adjusted for the same variables in Figure 4, as appropriate.

# **Supplementary Table 10. Subgroup analysis of associations between tea consumption and** **risk of macrovascular complications according to potential baseline risk factors(n=26,162).**

| Subgroups | Never in the past year | |  | Less than daily | |  | Daily | | *P*_interaction_ |
| --- | --- | --- | --- | --- | --- | --- | --- | --- | --- |
|  | Cases | HR |  | Cases | HR (95% CI) |  | Cases | HR (95% CI) |  |
| Age at baseline (years) |  |  |  |  |  |  |  |  | 0.701 |
| <50 | 626 | 1.00 |  | 817 | 0.99 (0.89,1.11) |  | 446 | 0.99 (0.86,1.15) |  |
| 50-59 | 1,718 | 1.00 |  | 1,607 | 1.07 (1.00,1.16) |  | 1,041 | 1.01 (0.92,1.11) |  |
| ≥60 | 2,738 | 1.00 |  | 2,070 | 1.05 (0.99,1.12) |  | 1,614 | 1.05 (0.97,1.13) |  |
| Sex |  |  |  |  |  |  |  |  | 0.164 |
| Female | 3,968 | 1.00 |  | 2,660 | 1.05 (0.99,1.11) |  | 1,275 | 1.08 (1.00,1.17) |  |
| Male | 1,114 | 1.00 |  | 1,834 | 1.03 (0.95,1.11) |  | 1,826 | 0.98 (0.90,1.07) |  |
| Region |  |  |  |  |  |  |  |  | 0.060 |
| Urban | 2,709 | 1.00 |  | 2,642 | 1.07 (1.01,1.13) |  | 1,726 | 1.08 (1.01,1.16) |  |
| Rural | 2,373 | 1.00 |  | 1,852 | 1.01 (0.94,1.08) |  | 1,375 | 0.96 (0.88,1.05) |  |
| Education |  |  |  |  |  |  |  |  | 0.094 |
| Middle school or higher | 1,756 | 1.00 |  | 2,191 | 1.10 (1.03,1.17) |  | 1,444 | 1.12 (1.03,1.22) |  |
| Primary school or lower | 3,326 | 1.00 |  | 2,303 | 1.00 (0.94,1.06) |  | 1,657 | 0.96 (0.89,1.04) |  |
| Smoking status |  |  |  |  |  |  |  |  | 0.644 |
| Current ^a^ | 758 | 1.00 |  | 1,218 | 1.04 (0.94,1.14) |  | 1,337 | 1.01 (0.91,1.12) |  |
| Not current | 4,324 | 1.00 |  | 3,276 | 1.05 (1.00,1.10) |  | 1,764 | 1.05 (0.98,1.12) |  |
| Alcohol consumption |  |  |  |  |  |  |  |  | 0.026 |
| Weekly or daily | 283 | 1.00 |  | 590 | 1.10 (0.95,1.28) |  | 624 | 0.93 (0.80,1.09) |  |
| Less than weekly | 4,799 | 1.00 |  | 3,904 | 1.04 (0.99,1.09) |  | 2,477 | 1.05 (0.99,1.11) |  |
| Fresh fruit consumption |  |  |  |  |  |  |  |  | 0.633 |
| ≥ 4 days/week | 1,240 | 1.00 |  | 1,304 | 1.09 (1.01,1.19) |  | 895 | 1.10 (0.99,1.22) |  |
| < 4 days/week | 3,842 | 1.00 |  | 3,190 | 1.02 (0.97,1.08) |  | 2,206 | 1.00 (0.93,1.07) |  |
| Physical activity (MET h/day) | |  |  |  |  |  |  |  | 0.027 |
| Male<5.65/Female<8.40 | 1,272 | 1.00 |  | 981 | 1.04 (0.94,1.14) |  | 774 | 1.14 (1.01,1.27) |  |
| Male5.65-12.19/Female8.40-11.69 | 1,399 | 1.00 |  | 1,206 | 1.08 (0.99,1.18) |  | 942 | 1.07 (0.96,1.19) |  |
| Male12.20-23.44/Female11.70-18.19 | 1,224 | 1.00 |  | 1,180 | 1.05 (0.96,1.14) |  | 812 | 1.02 (0.91,1.15) |  |
| Male≥23.45/Female≥18.20 | 1,187 | 1.00 |  | 1,127 | 1.00 (0.91,1.10) |  | 573 | 0.90 (0.79,1.02) |  |
| BMI (kg/m^2^) |  |  |  |  |  |  |  |  | 0.122 |
| ≥24.0 | 3,086 | 1.00 |  | 2,809 | 1.01 (0.95,1.06) |  | 1,914 | 1.02 (0.95,1.09) |  |
| <24.0 | 1,996 | 1.00 |  | 1,685 | 1.10 (1.02,1.18) |  | 1,187 | 1.04 (0.94,1.14) |  |
| WC (cm) |  |  |  |  |  |  |  |  | 0.055 |
| Male≥85/Female≥80 | 3,444 | 1.00 |  | 3,096 | 1.01 (0.96,1.07) |  | 2,114 | 1.00 (0.93,1.07) |  |
| Male<85/Female<80 | 1,638 | 1.00 |  | 1,398 | 1.12 (1.03,1.21) |  | 987 | 1.10 (0.99,1.22) |  |
| Prevalent hypertension |  |  |  |  |  |  |  |  | 0.692 |
| Yes | 3,458 | 1.00 |  | 2,962 | 1.03 (0.97,1.09) |  | 2,169 | 1.03 (0.96,1.10) |  |
| No | 1,624 | 1.00 |  | 1,532 | 1.07 (0.99,1.15) |  | 932 | 1.02 (0.92,1.13) |  |
| Diabetic treatment ^b^ |  |  |  |  |  |  |  |  | 0.140 |
| Yes | 1,816 | 1.00 |  | 1,641 | 1.04 (0.96,1.12) |  | 1,104 | 1.12 (1.02,1.23) |  |
| No | 3,266 | 1.00 |  | 2,853 | 1.07 (1.01,1.13) |  | 1,997 | 1.01 (0.95,1.09) |  |

Abbreviations: BMI: body mass index; CI, confidence interval; HR, hazard ratio; MET, metabolic equivalent of task (grouped by quartailes acording to sex); WC: waist circumference.

Values were obtained from Cox proportional hazards analysis. Except for the baseline stratifying variable, the model was adjusted for the same variables as in the model of Figure 3. *P*_interaction_, *P* from the likelihood ratio tests fro interaction.

^a^ Former smoker who had stopped smoking for illness was categorized into the current smoker.

^b^ Diabetic treatment includes insulin and/or oral hypoglycemic drugs.

# **Supplementary Table 11. Subgroup analysis of associations between tea consumption and risk of microvascular complications according to potential baseline risk factors (n=30,300).**

| Subgroups | Never in the past year | |  | Less than daily | |  | Daily | | *P*_interaction_ |
| --- | --- | --- | --- | --- | --- | --- | --- | --- | --- |
|  | Cases | HR |  | Cases | HR (95% CI) |  | Cases | HR (95% CI) |  |
| Age at baseline (years) |  |  |  |  |  |  |  |  | 0.729 |
| <50 | 112 | 1.00 |  | 191 | 1.08 (0.83,1.39) |  | 127 | 1.01 (0.75,1.38) |  |
| 50-59 | 297 | 1.00 |  | 336 | 0.94 (0.79,1.11) |  | 284 | 0.88 (0.72,1.08) |  |
| ≥60 | 404 | 1.00 |  | 374 | 0.99 (0.85,1.15) |  | 316 | 0.86 (0.72,1.03) |  |
| Sex |  |  |  |  |  |  |  |  | 0.209 |
| Female | 627 | 1.00 |  | 538 | 0.95 (0.83,1.07) |  | 368 | 0.94 (0.80,1.10) |  |
| Male | 186 | 1.00 |  | 363 | 1.03 (0.86,1.24) |  | 359 | 0.84 (0.69,1.03) |  |
| Region |  |  |  |  |  |  |  |  | 0.970 |
| Urban | 496 | 1.00 |  | 512 | 0.97 (0.85,1.10) |  | 378 | 0.89 (0.76,1.03) |  |
| Rural | 317 | 1.00 |  | 389 | 0.94 (0.79,1.12) |  | 349 | 0.85 (0.70,1.05) |  |
| Education |  |  |  |  |  |  |  |  | 0.640 |
| Middle school or higher | 343 | 1.00 |  | 463 | 1.04 (0.90,1.21) |  | 338 | 0.94 (0.79,1.12) |  |
| Primary school or lower | 470 | 1.00 |  | 438 | 0.92 (0.79,1.06) |  | 389 | 0.83 (0.70,0.99) |  |
| Smoking status |  |  |  |  |  |  |  |  | 0.349 |
| Current ^a^ | 129 | 1.00 |  | 256 | 1.04 (0.83,1.31) |  | 267 | 0.81 (0.63,1.03) |  |
| Not current | 684 | 1.00 |  | 645 | 0.96 (0.86,1.08) |  | 460 | 0.94 (0.81,1.08) |  |
| Alcohol consumption |  |  |  |  |  |  |  |  | 0.114 |
| Weekly or daily | 43 | 1.00 |  | 89 | 0.97 (0.66,1.42) |  | 98 | 0.66 (0.44,0.99) |  |
| Less than weekly | 770 | 1.00 |  | 812 | 0.98 (0.88,1.09) |  | 629 | 0.92 (0.81,1.05) |  |
| Fresh fruit consumption |  |  |  |  |  |  |  |  | 0.837 |
| ≥ 4 days/week | 206 | 1.00 |  | 250 | 0.99 (0.81,1.20) |  | 203 | 0.88 (0.70,1.11) |  |
| < 4 days/week | 607 | 1.00 |  | 651 | 0.95 (0.84,1.08) |  | 524 | 0.86 (0.74,1.00) |  |
| Physical activity (MET h/day) | |  |  |  |  |  |  |  | 0.760 |
| Male<5.65/Female<8.40 | 240 | 1.00 |  | 206 | 1.02 (0.83,1.25) |  | 179 | 1.08 (0.85,1.38) |  |
| Male5.65-12.19/Female8.40-11.69 | 235 | 1.00 |  | 253 | 0.93 (0.76,1.14) |  | 240 | 0.79 (0.63,1.00) |  |
| Male12.20-23.44/Female11.70-18.19 | 185 | 1.00 |  | 241 | 0.98 (0.79,1.22) |  | 198 | 0.95 (0.74,1.22) |  |
| Male≥23.45/Female≥18.20 | 153 | 1.00 |  | 201 | 0.95 (0.75,1.20) |  | 110 | 0.75 (0.55,1.02) |  |
| BMI (kg/m^2^) |  |  |  |  |  |  |  |  | 0.149 |
| ≥24.0 | 459 | 1.00 |  | 554 | 0.98 (0.86,1.12) |  | 412 | 0.80 (0.68,0.94) |  |
| <24.0 | 354 | 1.00 |  | 347 | 0.97 (0.82,1.14) |  | 315 | 1.01 (0.83,1.22) |  |
| WC (cm) |  |  |  |  |  |  |  |  | 0.978 |
| Male≥85/Female≥80 | 531 | 1.00 |  | 608 | 0.95 (0.83,1.07) |  | 493 | 0.85 (0.73,0.99) |  |
| Male<85/Female<80 | 282 | 1.00 |  | 293 | 0.99 (0.82,1.20) |  | 234 | 0.89 (0.71,1.12) |  |
| Prevalent hypertension |  |  |  |  |  |  |  |  | 0.002 |
| Yes | 557 | 1.00 |  | 540 | 0.88 (0.77,1.01) |  | 449 | 0.78 (0.67,0.91) |  |
| No | 256 | 1.00 |  | 361 | 1.17 (0.98,1.39) |  | 278 | 1.10 (0.89,1.35) |  |
| Diabetic treatment ^b^ |  |  |  |  |  |  |  |  | 0.101 |
| Yes | 487 | 1.00 |  | 504 | 0.94 (0.82,1.08) |  | 380 | 0.88 (0.75,1.03) |  |
| No | 326 | 1.00 |  | 397 | 1.06 (0.90,1.25) |  | 347 | 1.05 (0.87,1.26) |  |

Abbreviations: BMI: body mass index; CI, confidence interval; HR, hazard ratio; MET, metabolic equivalent of task (grouped by quartailes acording to sex); WC: waist circumference.

Values were obtained from Cox proportional hazards analysis. Except for the baseline stratifying variable, the model was adjusted for the same variables as in the model of Figure 4. *P*_interaction_, *P* from the likelihood ratio tests for interaction.

^a^ Former smoker who had stopped smoking for illness was categorized into the current smoker.

^b^ Diabetic treatment includes insulin and/or oral hypoglycemic drugs.
